# Supplementary material for: Defined extracellular ionic solutions to study and manipulate the cellular resting membrane potential
Source: Biol Open. 2020 Jan 14;9(1):bio048553. doi: 10.1242/bio.048553 (PMC6994931; doi:10.1242/bio.048553)
Supplement: Supplementary information [file biolopen-9-048553-s1.pdf]

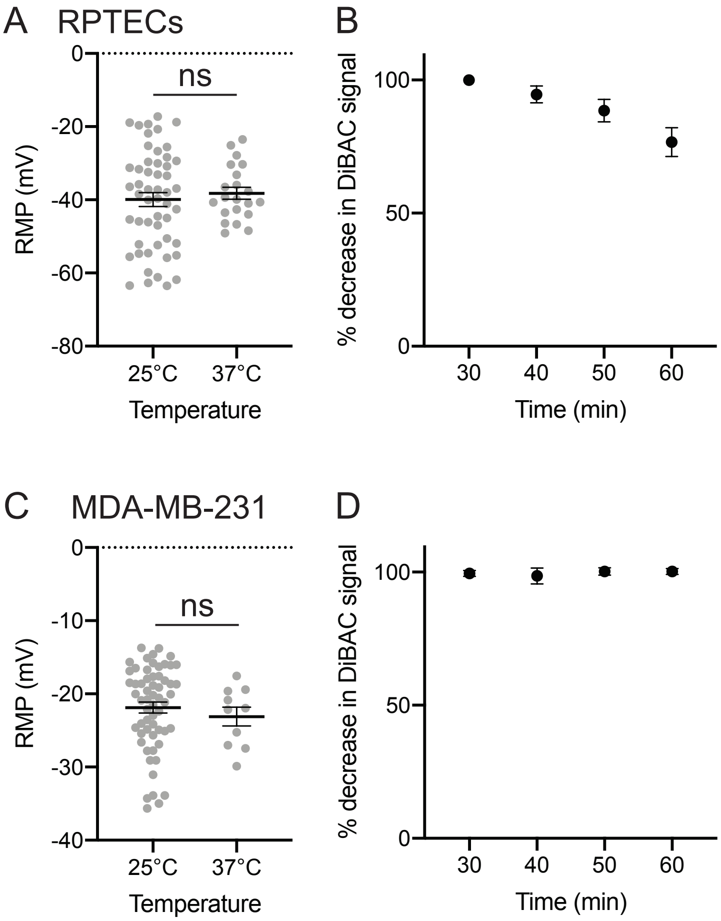

Figure S1: Stability of resting membrane potential and DiBAC signal. (A) RPTEC resting membrane potential (RMP) does not significantly vary at 25°C or 37°C when measured using patch clamp. **(B)** Change in DiBAC optical signal over 30 minutes of measurement (preceded by 30 minutes of incubation) in RPTECs. **(C)** MDA-MB-231 RMP does not significantly vary at 25°C or 37°C when measured using patch clamp. **(D)** Change in DiBAC optical signal over 30 minutes of measurement (preceded by 30 minutes of incubation) in MDA-MB-231s.

Biology Open • Supplementary information
